# The Presence of Ascites Affects the Predictive Value of HVPG on Early Rebleeding in Patients with Cirrhosis

**DOI:** 10.1155/2020/1329857

**Published:** 2020-11-24

**Authors:** Chuan Liu, Ruoyang Shao, Sining Wang, Guangchuan Wang, Lifen Wang, Mingyan Zhang, Yanna Liu, Mingkai Liang, Xiaoguo Li, Ning Kang, Jitao Wang, Dan Xu, Hua Mao, Chunqing Zhang, Xiaolong Qi

**Affiliations:** ^1^Department of Gastroenterology, Zhujiang Hospital, Southern Medical University, Guangzhou 510000, China; ^2^Department of Hematology, Nanfang Hospital, Southern Medical University, Guangzhou 510000, China; ^3^Department of Gastroenterology, Shandong Provincial Hospital, Shandong University, Jinan 250000, China; ^4^CHESS Center, Institute of Portal Hypertension, The First Hospital of Lanzhou University, Lanzhou 730000, China

## Abstract

**Background and Aims:**

Gastroesophageal variceal bleeding is a serious complication of portal hypertension in cirrhotic patients and could be predicted by hepatic venous pressure gradient (HVPG). However, whether the presence of ascites affects the prognostic value of HVPG for patients with acute variceal bleeding is still unknown. This retrospective study is aimed at investigating the influence of ascites on predictive performance of HVPG for early rebleeding in cirrhotic patients with acute variceal bleeding.

**Methods:**

In this retrospective study, a total of 148 patients with cirrhosis hospitalized for acute variceal bleeding who underwent HVPG measurement and endoscopic variceal ligation (EVL) for the prevention of rebleeding were included. The receiver operating characteristic curve (ROC) and logistical regression method were employed to analyze the predictive performance of HVPG for early rebleeding. The locally weighted scatterplot smoothing approach was adopted to assess the monotonicity between bleeding risk and HVPG.

**Results:**

A significantly higher HVPG level was observed in patients with early rebleeding compared to patients without rebleeding in the nonascites cohort. When using HVPG to predict early rebleeding, there was a lower area under curve in the ascites cohort compared to the nonascites cohort. HVPG was recognized as a risk factor for early rebleeding by a logistic regression model only in the nonascites cohort. An overall monotonicity in the trend of change in HVPG and risk for early rebleeding was observed in the nonascites cohort solely.

**Conclusion:**

The predictive value of HVPG for early rebleeding in patients with cirrhosis that developed acute variceal bleeding is hindered by the presence of ascites.

## 1. Introduction

Gastroesophageal variceal bleeding (GVB) is among the most serious complications of portal hypertension in patients with cirrhosis and even leads to death [1]. Hepatic venous pressure gradient (HVPG) is a potent prognostic factor for patients with cirrhosis [2–4] and has been widely recommended to predict the presence of ascites, hepatic encephalopathy, variceal bleeding and rebleeding, and bleeding-related death [4, 5]. An HVPG higher than 20 mmHg indicates a significantly higher risk of early rebleeding in patients with acute variceal bleeding (AVB) [3, 6–8].

Other than GVB, ascites is also commonly developed in patients with cirrhosis especially those with more advanced disease condition. The enhancing activation of renin-angiotensin-aldosterone system (RAAS) as the disease progresses is considered the main pathophysiological process to induce the generation of ascites [9]. Thus, compared to patients without ascites, patients with ascites have a generally worse liver function and more intense hyperdynamic condition that causes instability in hemodynamics [10]. Besides, patients with multiple decompensation events like variceal bleeding combined with ascites, namely, patients experiencing “further decompensation,” have worse prognosis than those with one decompensation event [11, 12]. Furthermore, ascites itself as a physical influential factor could also play a disturbing role during HVPG measurement. While it is clear that ascites could influence hemodynamics [9, 10], there still lacks evidence to show whether the presence of ascites affects the prognostic value of HVPG in patients with AVB. In this study, we aim to investigate the influence of ascites on the predictive performance of HVPG for early rebleeding in cirrhotic patients with AVB.

## 2. Patients and Methods

### 2.1. Study Population

In this study, a total of 148 consecutive patients with cirrhosis were retrospectively recruited from Shandong Provincial Hospital between October 2010 and August 2018. The inclusion criteria were as follows: (1) patients hospitalized for AVB with clinically and/or pathologically diagnosed cirrhosis; (2) patients who received octreotide and emergency endoscopic therapy as an initial intervention to stop the acute bleeding and then endoscopic variceal ligation (EVL) (combined with nonselective beta-blocker (NSBB), or alone when there was an NSBB contraindication) for preventing rebleeding; (3) patients who accepted transjugular HVPG measurement after the emergency endoscopic therapy and within 7 days before and 18 days after the first therapy among the following EVL sequence; and (4) patients who were followed up till the 42^nd^ day or developed rebleeding since accepting EVL. To avoid the influence of EVL on the accuracy of HVPG, patients who accepted HVPG measurement within 48 hours after EVL were excluded [13]. Early rebleeding was defined as rebleeding occurred within 42 days since EVL.

### 2.2. HVPG Measurement

HVPG measurements were performed using balloon catheters with a pressure transducer at the tip (Edwards Lifesciences, Irvine, Calif) complying with a reported protocol [14]. Before catheterization, a “zero measurement” was performed. The right hepatic vein was chosen for measurements whenever feasible. If stenosis or vein-to-vein shunt in the right hepatic vein was observed, the middle hepatic vein was chosen instead. The free hepatic venous pressure was measured close to the inferior vena cava (1-3 cm, approximately). Then, the balloon was inflated to occlude completely the chosen hepatic vein, and then, the wedged hepatic venous pressure was measured. Dynamic screening of each pressure was continued until the pressure reached a plateau, after which the values were recorded. All measurements were performed in triplicate at least, and the average value was taken as the result. HVPG was determined by subtracting the free hepatic venous pressure from the wedged hepatic venous pressure.

### 2.3. Statistical Analysis

Continuous variables were shown as the mean and standard deviation (SD) or median and interquartile range (IQR). Categorical variables were shown as the number and frequency (%). The Mann–Whitney test was used to compare HVPG between nonearly rebleeding and early rebleeding in the ascites and nonascites subgroups. The receiver operating characteristic curve (ROC) was used to evaluate the predictive performance of HVPG for early rebleeding in the nonascites cohort and the ascites cohort, respectively. Univariate and multivariate logistic regression models were employed to calculate odds ratio (OR) and *P* value of HVPG and other potential risk stratification factors for rebleeding. For a multivariate logistic regression model, platelet (PLT), albumin (ALB), and HVPG were included. The locally weighted scatterplot smoothing (LOWESS) approach was adopted to assess the monotonicity between bleeding risk and HVPG in patients with and without ascites. All levels of significance were set at a two-sided 5% level. All analyses were performed using SPSS 22.0 IBM (IBM Corp., Armonk, NY) and R 3.5.3 (R Project for Statistical Computing, Vienna, Austria).

## 3. Results

### 3.1. Patients

A total of 148 patients meeting the inclusion and exclusion criteria were included, of which 106 patients received either propranolol or carvedilol combined with EVL. Patients included were followed up until at least the 42^nd^ day or developed rebleeding since EVL. Early rebleeding occurred in 15 out of 148 patients (10.1%). Clinical characteristics of the studied cohorts are summarized in Table 1.

### 3.2. HVPG Remains Stable in Patients with Ascites Who Developed Early Rebleeding

**Figure 1:**
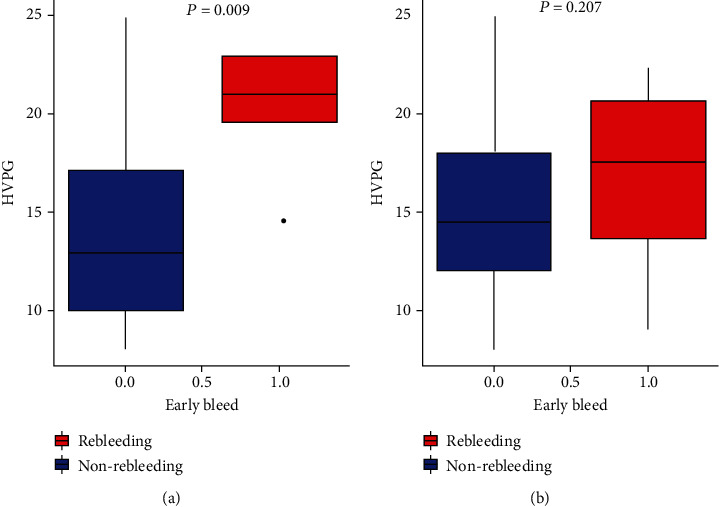
Comparisons of HVPG in patients with and without early rebleeding in (a) the nonascites cohort and (b) the ascites cohort. HVPG: hepatic venous pressure gradient.

During follow-up, 10 out of 79 patients with ascites (ascites cohort) and 5 out of 69 patients without ascites (nonascites cohort) experienced early rebleeding. We compared the HVPG level between patients with and without early rebleeding in both cohorts. In the nonascites cohort, a significantly higher HVPG level was observed in patients experienced early rebleeding compared to those did not (21.00 mmHg vs. 13.00 mmHg, *P* = 0.009) (Figure 1(a)). However, there was no significant difference in the HVPG level between patients with and without early rebleeding in the ascites cohort (Figure 1(b), median, 17.50 (12.34-21.00) mmHg vs. 14.50 (12.00-18.00) mmHg, *P* = 0.207).

### 3.3. Ascites Affects the Predictive Value of HVPG for Early Rebleeding

**Figure 2:**
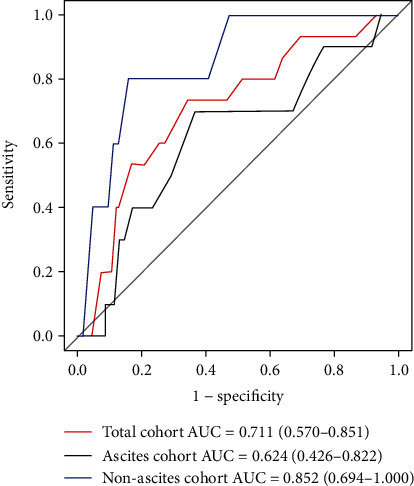
ROC curve of HVPG for predicting early rebleeding in the total cohort, the ascites cohort, and the nonascites cohort. AUC: area under the ROC curve.

We used the area under the ROC curve (AUC) to assess whether the presence of ascites affects the predictive value of HVPG on early rebleeding. The ROC curves were plotted for the whole cohort, the ascites cohort, and the nonascites cohort (Figure 2). AUC values of HVPG for predicting early rebleeding showed a tendency to decrease in the three cohorts (AUC: 0.711 (0.570-0.851), 0.852 (0.694-1.000), and 0.624 (0.426-0.822) for whole, nonascites, and ascites cohorts, respectively) (Figure 2).

### 3.4. The Impact of HVPG on the Risk of Early Rebleeding Is Different in Patients with and without Ascites

To investigate the risk factors for early rebleeding in patients with and without ascites, univariate and multivariate logistic regression analysis were performed. In the nonascites cohort, HVPG was recognized as the only statistically significant risk factor with ORs of 1.350 (*P* = 0.020, univariate) and 1.350 (*P* = 0.029, multivariate) (Table 2). However, in the ascites cohort, HVPG failed to manifest a significant impact on the risk of early rebleeding with ORs of 1.089 (*P* = 0.253, univariate) and 1.073 (*P* = 0.380, multivariate) (Table 3).

**Figure 3:**
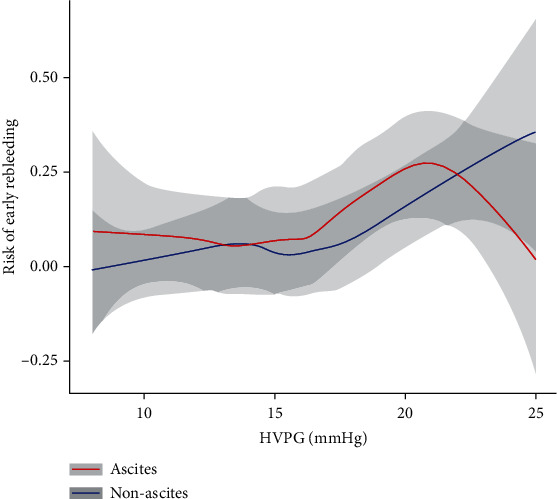
LOWESS curve for assessing the trend of risk for early rebleeding on HVPG in the ascites cohort and nonascites cohort.

It is generally believed that the higher the HVPG of cirrhotic patients, the higher risk for rebleeding they suffer. So we believe that if the HVPG level is a risk factor of early rebleeding in a certain population, there should be an overall monotonicity in the trend of change in HVPG and the risk for early rebleeding. Therefore, we employed the LOWESS approach to generate a fitting curve that reflected the overall trend of change in HVPG and risk for early rebleeding in the ascites cohort and the nonascites cohort, respectively, in order to assess their monotonicity. As shown in Figure 3, an overall monotonicity was observed in the nonascites cohort but not in the ascites cohort.

## 4. Discussion

HVPG could filter the influence of the central venous system and abdominal pressure and is widely accepted as an accurate index for assessing portal hypertension [15–17]. It has been proved to be a potent and versatile prognostic factor in cirrhotic portal hypertension. HVPG ≥ 10 mmHg is regarded as the threshold for the occurrence of decompensation and is thus called clinically significant portal hypertension. Patients with clinically significant portal hypertension face significantly higher risks of developing varices, bleeding, other decompensation events, and hepatocellular carcinoma [2, 18, 19]. Patients with an HVPG ≥ 16 mmHg suffer from higher mortality [20–22] and bleeding risk [7, 23]. An HVPG above 20 mmHg is strongly predictive of failure to control bleeding, early rebleeding, and hemorrhage-related death [3, 24].

It is intuitive and generally successful to stratify bleeding risk using the stable portal pressure reflector, HVPG, based on the direct correlation between the elevation of portal pressure and risk of varices bleeding. However, there still exist confounding factors affecting either the accuracy of HVPG measurement or its capability to indicate the actual bleeding risk in patients with cirrhosis, especially those with more complex disease conditions, like patients with ascites.

In patients with cirrhosis, the presence of ascites is the consequence of the activation of RAAS initiated by portal hypertension. Approximately 60% of cirrhotic patients develop ascites in 10 years since diagnosis [25], and ascites is the first decompensation event in most patients [26, 27].

In studies that support the role for an HVPG higher than 20 mmHg to indicate a higher risk of treatment failure or early rebleeding, none of them performed subgroup analysis for patients with and without ascites [3, 6–8, 28]. However, as stated above, patients with ascites have generally more advanced disease condition and poorer liver function and are therefore more easily to develop endothelial dysfunction [29]. Under these circumstances, HVPG could not accurately reflect the portal pressure for it actually represents the pressure of the hepatic sinusoid. Besides, patients with ascites are in a more intense hyperdynamic state and with more unstable hemodynamics [10, 30]. These patients, even with relatively low HVPG, may suffer from higher risks of rapid increment of HVPG and exacerbation of disease that results in worse clinical outcomes, compared to patients with similar HVPG but without ascites. A significantly higher mortality was observed in patients with ascites compared to patients without any decompensation events, and the result was also similar when comparing patients with ascites and experienced bleeding to those who experienced bleeding but without other decompensation events [11, 12]. Also, although the elevation of portal pressure is considered the dominant factor of bleeding, the more complex condition in patients with ascites inevitably adds more influential factors and thus hinders the predictive performance of the single predictor, HVPG. Additionally, although HVPG could filter the influence of the central venous system and abdominal pressure theoretically, the measurement error introduced by respiratory cycle cannot be eliminated [31].

One possible solution to improve the early rebleeding-predictive performance is to combine HVPG with other clinical indicators to develop an extended predictive model. In a meta-analysis that included 118 studies, Child-Pugh, encephalopathy, hepatocellular carcinoma, bleeding, creatinine, prothrombin time, albumin, azotemia, ascites, and bilirubin were shown to be frequently used statistically significant prognostic parameters in patients with decompensated cirrhosis [32]. By introducing other clinical indicators, a model that covers different factors that influence clinical outcome from different aspects could be developed. A multiple factor model may be able to reflect the disease condition of patients in a more comprehensive manner, resulting in possible improvement in predictive performance. However, the more indicators included in a model, the less easy-to-use the model will be. Another possible attempt is to track the change of HVPG after acute bleeding. As reported by Ready et al., acute bleeding patients who did not develop early rebleeding showed an overall decreasing trend of HVPG after the acute phase [28]. Overcoming the invasiveness and high cost of extra HVPG measurements, the emerging techniques for noninvasive prediction of portal pressure have achieved high accuracy using routine clinical data [1, 33–35]. These serum- or imaging-based methods may provide additional data that benefit our decision. Nevertheless, the sensitivity of these methods to the short-term change of portal pressure remains to be tested before being applied for dynamic monitoring.

Our study for the first time investigated the influence of ascites on the predictive value of HVPG for early rebleeding in cirrhotic patients with AVB. Yet, there are also several limitations. First, this study is a retrospective study including cases from a single center, which may be a possible source of bias. Second, subgroup analysis was not performed for patients with ascites of different intensities due to lack of original data. Third, not all the patients included received NSBB, and this heterogeneity may also be a source of bias. Fourth, patients were followed up for only 42 days, so no data on other events could be provided.

In summary, we found that patients with early rebleeding have a higher HVPG than those who did not in the nonascites cohort, but not in the ascites cohort. When using HVPG to predict early rebleeding, the AUC in the ascites cohort was significantly lower comparing to the nonascites cohort and the whole cohort. HVPG was recognized as a risk factor for early rebleeding in the nonascites cohort but not in the ascites cohort. An overall monotonicity in the trend of change in HVPG and risk for early rebleeding was observed in the nonascites cohort only using the LOWESS approach. Taking together, these findings suggested that the predictive value of HVPG for early rebleeding in patients with cirrhosis that developed AVB is hindered by the presence of ascites.

## Figures and Tables

**Table 1:** Clinical characteristics of the studied patients.

Variables	Patients (*n* = 148)	Ascites group (*n* = 79)	Nonascites group (*n* = 69)	*P*
Age (y), median (IQR)	51.5 (15.75)	53.0 (16.00)	50.0 (13.50)	0.071
Gender, *n* (%)				0.607
Male	46 (31.1)	53 (67.1)	20 (29.0)	
Female	102 (68.9)	26 (32.9)	49 (71.0)	
AST (IU/L), median (IQR)	33.5 (20.5)	34.0 (19.0)	33.0 (24.0)	0.745
ALT (IU/L), median (IQR)	25.0 (15.8)	25.0 (19.0)	25.0 (14.0)	0.917
PLT (10^9^/L), median (IQR)	71.5 (72.5)	66.0 (71.0)	83.0 (78.0)	0.118
TBIL (*μ*mol/L), median (IQR)	19.9 (10.7)	20.5 (12.1)	18.9 (9.85)	0.138
ALB (g/L), median (IQR)	33.4 (7.4)	31.2 (7.7)	34.7 (7.4)	<0.001
INR, median (IQR)	1.21 (0.25)	1.24 (0.25)	1.20 (0.16)	0.019
Accepting NSBB, *n* (%)	106 (71.6)	48 (60.76)	58 (84.06)	0.002
Ascites, *n* (%)	79 (53.4)	NA	NA	NA
Early rebleeding, *n* (%)	15 (10.1)	10 (12.66)	5 (7.25)	0.414
HVPG (mmHg), mean (SD)	15.0 (4.66)	15.46 (4.52)	14.44 (4.79)	0.158
Child-Pugh class, *n* (%)				<0.001
Child A	62 (41.9)	14 (17.72)	48 (69.57)	
Child B	73 (49.4)	53 (67.09)	20 (28.99)	
Child C	13 (8.8)	12 (15.19)	1 (1.45)	
Etiology, *n* (%)				0.165
Viral	87 (58.7)	53 (67.09)	34 (49.28)	
Alcoholic	16 (10.8)	8 (10.13)	8 (11.59)	
Autoimmunogenic	10 (6.7)	5 (6.33)	5 (7.25)	
Cholestatic	5 (3.4)	1 (1.27)	4 (5.80)	
Other	30 (20.3)	12 (15.19)	18 (26.09)	

AST: aspartate aminotransferase; ALT: alanine aminotransferase; PLT: platelets; TBIL: total bilirubin; INR: international normalized; ALB: albumin; NSBB: nonselective beta-blocker; MELD: Model of End-stage Liver Disease; HVPG: hepatic venous pressure gradient; y: years; IQR: interquartile range.

**Table 2:** Univariate and multivariate logistic regression analysis in the nonascites cohort.

Variable	Univariate		Multivariate	
	OR	*P* value	OR	*P* value
Child-Pugh score	0.543 (0.061-4.807)	0.583		
HVPG	1.350 (1.049-1.737)	0.020	1.350 (1.032-1.765)	0.029
AST	0.988 (0.932-1.047)	0.681		
ALT	0.9996 (0.954-1.040)	0.863		
ALB	1.066 (0.889-1.278)	0.490		
TBIL	0.998 (0.979-1.018)	0.850		
PLT	0.997 (0.983-1.010)	0.614		
INR	6.989 (0.061-804.738)	0.422	1.014 (0.002-438.036)	0.996

HR: hazard ratio; HVPG: hepatic venous pressure gradient; AST: aspartate aminotransferase; ALT: alanine aminotransferase; ALB: albumin; TBIL: total bilirubin; PLT: platelets; INR: international normalized ratio.

**Table 3:** Univariate and multivariate logistic regression analysis in the ascites cohort.

Variable	Univariate		Multivariate	
	OR	*P* value	OR	*P* value
Child-Pugh score	3.278 (0.952-11.289)	0.060		
HVPG	1.089 (0.941-1.261)	0.253	1.073 (0.917-1.255)	0.380
AST	0.980 (0.942-1.019)	0.310		
ALT	0.969 (0.916-1.025)	0.267		
ALB	0.965 (0.864-1.078)	0.531		
TBIL	1.026 (0.993-1.061)	0.125		
PLT	0.980 (0.957-1.004)	0.096		
INR	17.052 (1.014-286.888)	0.049	14.364 (0.825-250.056)	0.068

HR: hazard ratio; HVPG: hepatic venous pressure gradient; AST: aspartate aminotransferase; ALT: alanine aminotransferase; ALB: albumin; TBIL: total bilirubin; PLT: platelets; INR: international normalized ratio.

## Data Availability

The data that support the findings of this study are available from the corresponding author upon reasonable request.
